# Overcoming Scale Variations and Occlusions in Aerial Detection: A Context-Aware DEIM Framework

**DOI:** 10.3390/s26010147

**Published:** 2025-12-25

**Authors:** Xinhao Chang, Xuejuan Wang, Kefeng Li

**Affiliations:** 1 School of Rail Transportation, Shandong Jiaotong University, Jinan 250357, China; changan4216@163.com; 2 School of Information Science and Electrical Engineering, Shandong Jiaotong University, Jinan 250357, China; 205073@sdjtu.edu.cn

**Keywords:** object detection, UAV-OD, DEIM, small objects

## Abstract

Object detection in Unmanned Aerial Vehicle (UAV) imagery has gained significant traction in applications such as railway inspection and waste management. While emerging end-to-end detectors like DEIM show promise, they often struggle with weak feature responses and spatial misalignment in aerial scenarios. To address these issues, this paper proposes SCA-DEIM, a context-aware real-time detection framework. Specifically, we introduce the Adaptive Spatial and Channel Synergistic Attention (ASCSA) module, which refines existing attention paradigms by transitioning from a static gating mechanism to an active signal amplifier. Unlike traditional designs that impose rigid bounds on feature responses, this improved architecture enhances feature extraction by dynamically boosting the saliency of faint small-target signals amidst complex backgrounds. Furthermore, drawing inspiration from infrared small object detection, we propose the Cross-Stage Partial Shifted Pinwheel Mixed Convolution (CSP-SPMConv). By synergizing asymmetric padding with a spatial shift mechanism, this module effectively aligns receptive fields and enforces cross-channel interaction, thereby resolving feature misalignment and scale fusion issues. Comprehensive experiments on the VisDrone2019 dataset demonstrate that, compared with the baseline model, SCA-DEIM achieves improvements of 1.8% in Average Precision (AP), 2.3% in AP for small objects ($AP_{s}$), and 2.0% in AP for large objects ($AP_{l}$), while maintaining a competitive inference speed. Notably, visualization results under different illumination conditions demonstrate the strong robustness of the model. In addition, further validation on both the UAVVaste and UAVDT datasets confirms that the proposed method effectively enhances the detection performance for small objects.

## 1. Introduction

With the advent of Unmanned Aerial Vehicle (UAV) technology, UAV object detection (UAV-OD) has emerged as a prominent research subfield within the broader domain of object detection [1]. UAVs equipped with cameras, leveraging their high mobility, deployment flexibility, and expansive field of view, have demonstrated immense application potential across a multitude of domains, such as precision agriculture, infrastructure inspection, disaster search and rescue, public safety surveillance, and intelligent traffic management [2]. However, in contrast to conventional object detection tasks, aerial imagery captured by UAVs often presents a unique set of challenges. These include drastic scale variations, a large number of small and occluded objects, diverse illumination conditions, and image blur induced by the UAV’s motion [3]. Consequently, the research and development of object detection algorithms for UAV platforms, capable of efficiently and accurately identifying small objects, are of significant theoretical importance and practical value [4].

DETR [5] pioneered the end-to-end object detection paradigm by integrating a Transformer [6] architecture, eliminating the need for hand-crafted components like Non-Maximum Suppression (NMS). Despite its innovative one-to-one (O2O) matching strategy, the original DETR faces inherent challenges in optimization efficiency and small object localization. Specifically, its convergence on the COCO dataset [7] is computationally expensive, often requiring significantly longer training schedules compared to conventional detectors like Faster R-CNN [8]. Furthermore, its attention mechanism, while powerful for global context, initially struggled to capture fine-grained details required for small targets.

To address the issues inherent in DETR, Huang et al. proposed a novel training framework, DETR with Improved Matching (DEIM) [9]. This framework employs a Dense one-to-one (O2O) matching strategy and a Matching-Aware Loss (MAL) to alleviate the problems of sparse supervision and low-quality matches within the DETR model. Specifically, it intensifies supervision by generating more positive samples using mosaic and mixup augmentations, and subsequently optimizes the matches of varying quality produced by these enhancement techniques through MAL. The combination of the Dense O2O strategy and MAL effectively accelerates the model’s convergence speed. Moreover, its convergence speed and accuracy on the COCO dataset have surpassed those of YOLOv8 [10], YOLOv9 [11], and YOLOv10 [12].

**Figure 1:**
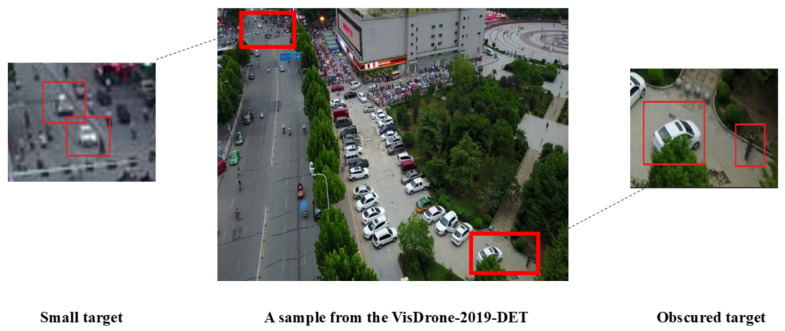
Small and occluded targets in the VisDrone 2019 dataset.

Despite DEIM’s superior performance in natural scenes, its direct application to UAV object detection faces distinct challenges inherent to the aerial environment, as illustrated in Figure 1. First, high-altitude capture results in drastic scale variations, where targets often occupy extremely low resolutions (e.g., fewer than 16×16 pixels). This lack of spatial detail weakens the semantic feature response, causing DEIM’s sparse attention mechanism to fail in locking onto faint targets. Second, UAV imagery frequently contains high-density object clusters and severe inter-class occlusions (e.g., crowded pedestrians or traffic jams). These complex spatial distributions can confuse DEIM’s one-to-one matching strategy, leading to missed detections or unstable convergence. Finally, environmental factors such as motion blur induced by UAV vibration and diverse illumination conditions introduce significant background noise. This noise exacerbates spatial misalignment, as the standard convolution operations in DEIM struggle to extract robust geometric features from blurred boundaries.

Upon an in-depth analysis of the DEIM architecture, we identified two primary deficiencies: feature extraction and feature fusion.

Regarding feature extraction, the backbone of DEIM utilizes a channel-only attention module, the Efficient Squeeze-Excitation (ESE) Attention, to achieve a lightweight design. This, however, comes at the cost of sacrificing critical spatial information. Consequently, the model’s ability to perceive spatially sensitive targets (such as small and occluded objects) is severely compromised. With respect to feature fusion, DEIM employs symmetric, VGG-style standard convolutions for feature integration. This approach treats all pixels within its receptive field symmetrically, rendering it unable to effectively differentiate orientation-specific features. This inefficiency in capturing anisotropic structural information, such as edges and textures, leads to suboptimal detection capability for small and occluded objects. Beyond addressing scale variations, maintaining feature consistency is critical. However, deep networks risk signal degradation. As analyzed by Zhao et al. [13], nonlinear error accumulation can sever the correlation between inputs and high-level representations over depth. In UAV scenarios, this implies that without specific preservation mechanisms, the faint cues of small objects are prone to being overwhelmed by cumulative noise.

To overcome these challenges, this paper presents SCA-DEIM, a refined, lightweight architecture built upon the DEIM model. The framework introduces two novel modules, Adaptive Spatial and Channel Synergistic Attention (ASCSA) and Cross-Stage Partial Shifted Pinwheel Mixed Convolution (CSP-SPMConv), to remedy the aforementioned deficiencies in feature extraction and fusion, respectively.

Specifically, ASCSA is an adaptive spatial and channel synergistic attention module that combines axis-aware pooling with multi-scale convolution to augment spatial information within high-level semantic features. It also utilizes channel self-attention to dynamically model complex inter-channel relationships. In contrast to the channel-only ESE mechanism in the DEIM backbone, ASCSA preserves the location and structure of small objects in the spatial dimension and dynamically correlates context along the channel dimension, thereby enhancing the capability to extract features from small and occluded targets.CSP-SPMConv is a multi-scale shifted pinwheel mixed convolution module based on the CSP architecture. Unlike the symmetric, VGG-style standard convolutions used in DEIM, the distinguishing features of CSP-SPMConv are its use of asymmetric padding and the concept of feature shifting. This design enables feature mixing across spatial and channel dimensions with negligible additional computational cost, thereby significantly enhancing the model’s feature representation power and achieving more effective multi-scale feature fusion. The design philosophy and implementation of these two modules are detailed in the Related Work and Methodology sections of this paper.

1. We propose the Adaptive Spatial and Channel Synergistic Attention (ASCSA) module. Unlike the standard SCSA whose weights are strictly constrained to the (0, 1) interval, ASCSA introduces a dynamic amplification mechanism that allows attention weights to exceed 1, enabling the network to actively enhance faint small-object cues. In addition, its decoupled spatial-channel synergy strategy effectively captures global semantics while reducing computational redundancy.
2. We introduce an efficient convolutional module, Cross-Stage Partial Shifted Pinwheel Mixed Convolution (CSP-SPMConv). By leveraging asymmetric padding and feature shifting, this module achieves a more effective multi-scale fusion of the extracted features.
3. The synergistic operation of these modules concurrently achieves both a lightweight design and significant performance improvements. Experimental results on the VisDrone2019 dataset [14] demonstrate that, compared to the baseline, SCA-DEIM remains lightweight while achieving performance gains of 1.8% in AP, 2.3% in ${\mathrm{AP}}_{s}$, and 2.0% in ${\mathrm{AP}}_{l}$. A series of ablation studies further confirms that ASCSA and CSP-SPMConv possess a significant synergistic relationship.

## 2. Related Work

### 2.1. End-to-End Object Detection

The advent of DETR (Detection Transformer) has garnered significant enthusiasm within the research community for end-to-end object detection models. In contrast to conventional detectors, it successfully performs predictions without relying on anchors or hand-crafted components, achieving commendable results. Nevertheless, DETR exhibits suboptimal performance on small objects, and its convergence speed on the COCO dataset is considerably slower than contemporaneous methods. Despite these limitations, it did not detract from its potential as a new paradigm in the field of object detection.

In recent years, researchers have continually explored enhancements for DETR. Deformable DETR, proposed by Zhu et al. [15], utilized deformable convolution to effectively mitigate the slow convergence and high computational complexity issues of the original DETR [16]. DAB-DETR, introduced by Liu et al. [17], focused on the queries by directly learning anchor boxes as queries, thereby improving both convergence speed and accuracy. DN-DETR employed a denoising strategy to avoid the matching instability caused by the Hungarian algorithm, which accelerated the training process [18]. Inspired by these three predecessors, DINO further advanced the DETR family by refining the denoising training, optimizing query initialization, and leveraging deformable attention mechanisms [19].

To remedy the real-time limitations of the DETR series, Zhao et al. proposed RT-DETR, the first real-time end-to-end object detector [20]. It improves intra-scale interaction and cross-scale fusion through a refined encoder and designs minimal uncertainty queries to enhance initial query quality, enabling the model to balance speed and accuracy effectively. While these models have surpassed the original DETR and most YOLO series methods in terms of accuracy and speed, their computational overhead (GFlops) and parameter counts (Params) are substantially higher than those of the YOLO series, leading to increased training costs. D-Fine, proposed by Peng et al., successfully introduced a lightweight design to real-time end-to-end detectors while still maintaining excellent accuracy and speed [21].

While the aforementioned detection methods have achieved substantial advancements in accuracy and speed, small object detection within the domain of UAV object detection remains a pressing and unresolved challenge. Drawing inspiration from RT-DETR, we identified that DEIM still possesses considerable room for improvement, particularly in enhancing its local feature extraction and multi-scale feature fusion capabilities.

### 2.2. Spatial-Channel Synergistic Attention and Multi-Scale Fusion Technique

Feature extraction aims to obtain discriminative representations from input images. Traditional CNN models, such as VGG [22], ResNet [23], and HGNet [24], primarily extract features through layer-by-layer convolution. However, simple convolutions often struggle to preserve critical fine-grained information in complex aerial scenes. To address this issue, various attention mechanisms have been proposed, such as ECA [25], CBAM [26], SAA [27], and SCSA [28], which effectively achieve spatial-channel synergy. Nevertheless, most existing attention mechanisms—including SCSA—rely on the Sigmoid gating function, which strictly constrains the output values to the (0, 1) range. Although this gating facilitates feature selection, it limits the model’s ability to amplify weak feature signals of small objects, which is crucial for UAV detection tasks where targets are often faint and easily overwhelmed by background noise. In addition, Du et al. proposed TSD-YOLO [29], which incorporates an SPD module, selective kernel attention, and the WIoUv3 loss to enhance multi-scale robustness and alleviate background interference in traffic-sign detection, offering useful insights for UAV object detection as well.

Effectively fusing features from different levels is critical for detecting objects across various scales. Originally designed for infrared small target detection, Pinwheel-Shaped Convolution (PConv) [30] utilizes asymmetric padding to expand the receptive field. Given the morphological similarity between infrared targets and distant aerial objects (e.g., lacking detailed texture), this design effectively matches the distribution of small targets in UAV imagery. However, a critical limitation of PConv is that its parallel branches operate independently before the final fusion stage. Specifically, due to asymmetric padding, the feature maps from different branches correspond to spatially disjoint receptive field centers, resulting in spatial misalignment. PConv simply concatenates these misaligned features, forcing the network to implicitly infer spatial correspondence, which is inefficient for tiny targets. In contrast, our proposed CSP-SPMConv incorporates a `Shift Channel Mix’ mechanism to explicitly address this issue. Unlike PConv or simple channel shuffle operations, our method cyclically shifts the spatial features from different branches to geometrically realign them to a unified coordinate (i,j). This ensures that the subsequent fusion layer processes spatially coherent context from all four directions, transforming the `independent extraction’ of PConv into a `synergistic aggregation’ process.

## 3. Methods

### 3.1. Model Architecture of SCA-DEIM

**Figure 2:**
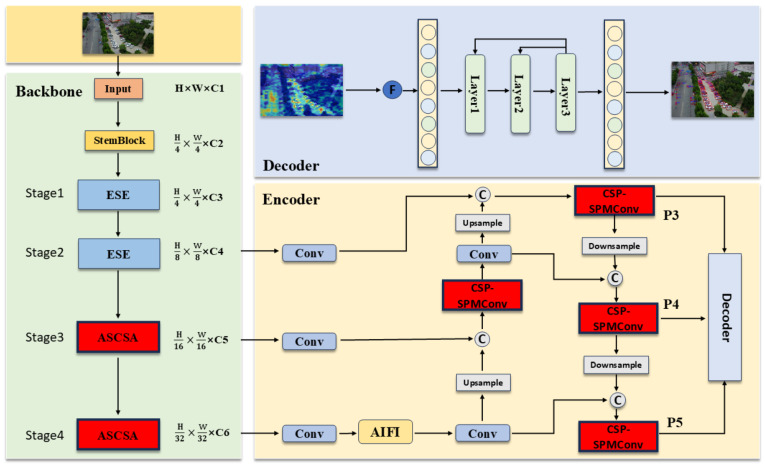
Architecture of SCA-DEIM.

As illustrated in Figure 2, the architecture of SCA-DEIM is partitioned into three principal components: the Backbone, the Encoder, and the Decoder. Within the Backbone, we introduce the Adaptive Spatial and Channel Synergistic Attention (ASCSA) module into the high-level semantic feature extraction stages, while retaining the original ESE (Effective Squeeze and Excitation) attention mechanism in the earlier stages. In the Encoder, we employ the Cross-Stage Partial Shifted Pinwheel Mixed Convolution (CSP-SPMConv) module to perform multi-scale feature fusion. This is designed to further enhance the features extracted by ASCSA.

### 3.2. Adaptive Spatial and Channel Synergistic Attention Module

**Figure 3:**
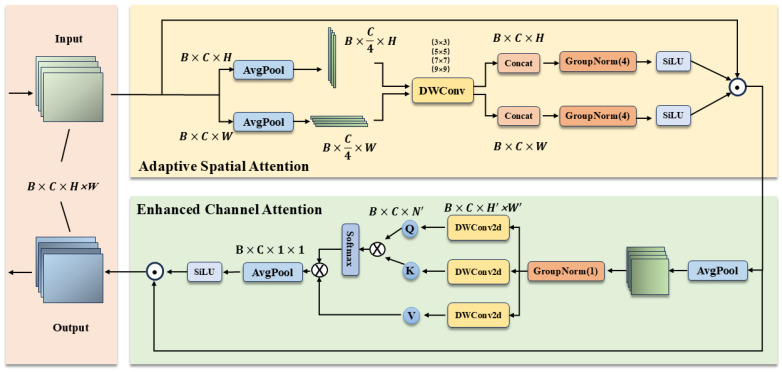
Architecture of the ASCSA module, where DWConv denotes Depth-wise Convolutions.

In UAV-based object detection, small targets typically occupy limited pixel areas, resulting in extremely weak feature responses that are easily overwhelmed by background noise. Although the standard SCSA effectively models spatial-channel synergy, we identify a critical limitation in its design: the reliance on the Sigmoid function confines attention weights strictly to the (0,1) interval. Consequently, SCSA functions solely as a “soft gate” that retains or suppresses information but lacks the capability to amplify critical yet faint target signals.To overcome this limitation, we propose the Adaptive Spatial and Channel Synergistic Attention (ASCSA). As illustrated in Figure 3, this module introduces two key innovations over the standard SCSA:

1. Dynamic Signal Amplification via Unbounded Activation: To overcome the signal attenuation inherent in Sigmoid’s (0,1) constraint, ASCSA employs the SiLU function, defined as f(x)=x·σ(x). Unlike the rigid “soft gate” of Sigmoid, SiLU possesses an unbounded positive domain ([−0.278,+∞)), functioning as an adaptive gain controller. This enables the module to simultaneously suppress background noise (x<0) and actively amplify faint target features (x>0) with attention weights exceeding unity, significantly enhancing the detectability of small and occluded objects.
2. Efficient Decomposition: It employs a decomposed spatial and channel attention technique, which not only minimizes parameter overhead but also captures global semantics and contextual information more effectively.

This mechanism consists of an Adaptive Spatial Attention component and an Enhanced Channel Attention component, and the specific formulations are as follows:

In the Adaptive Spatial Attention component, spatial operations are first performed on the input feature $X\in R^{C\times H\times W}$. For each channel, the spatial dimensions—height (*H*) and width (*W*)—are decomposed. Through average pooling, $z^{h}\in R^{C\times H\times 1}$ and $z^{w}\in R^{C\times 1\times W}$ are obtained.

(1) $$X_{H}=\frac{1}{W}\sum_{w=1}^{W}X\left[:,:,h,w\right],X_{W}=\frac{1}{H}\sum_{H=1}^{H}X\left[:,:,h,w\right]$$

Subsequently, we decompose each feature into *K* independent sub-features, each with C/K channels, to facilitate the learning of diverse spatial distributions and contextual information. To effectively capture the distinct semantic spatial structures within each sub-feature, we employ Depth-wise 1D Convolutions [31]. These convolutions employ kernel sizes of 3, 5, 7, and 9. Specifically, the small kernels (3 and 5) extract local low-level semantics, whereas the large kernels (7 and 9) capture global high-level semantics. To align with these four distinct receptive field scales, we set the group number *K* to 4. This choice is driven by the fact that a single sub-feature fails to encompass all semantic dimensions, while excessive sub-features risk diluting feature expressiveness and increasing computational overhead. Therefore, K=4 represents an optimal trade-off for balancing multi-level semantics. This configuration enables the ASCSA module to parallelly capture spatial contextual information across four scales with exceptional efficiency, thereby significantly enhancing the feature representation capability of its spatial attention component.

(2) $$X_{H}=\left[L_{h},G_{h_{s}},G_{h_{m}},G_{h_{l}}\right],X_{W}=\left[L_{w},G_{w_{s}},G_{w_{m}},G_{w_{l}}\right]$$

The sub-features processed by the depth-wise convolutions are then passed through the SiLU activation function for non-linear transformation, which serves to maintain non-linear expressiveness while providing smoother gradient variations. The spatial attention in each dimension is computed as follows:

(3) $$\begin{array}{c} \sigma \left(x\right)=\frac{1}{1+e^{-x}},\;\mathrm{SiLU}\left(x\right)=x\cdot \sigma \left(x\right)=\frac{x}{1+e^{-x}} \end{array}$$

(4) $$\begin{array}{c} G_{\tilde{x}}=\mathrm{cat}\left(DWconv\left(L_{x}\right),\;DWconv\left(G_{x_{s}}\right),\;DWconv\left(G_{x_{m}}\right),\;DWconv\left(G_{x_{l}}\right)\right),\\ A_{x}=\mathrm{SiLU}\left({\mathrm{Norm}}_{x}\left(G_{\tilde{x}}\right)\right),\;x\in \left\{h,w\right\} \end{array}$$

Finally, we apply the computed spatial attention weights to the input feature X to obtain the spatially enhanced feature $X^{\prime }$:

(5) $$X^{\prime }=XA_{h}A_{w}$$

In the Enhanced Channel Attention component, first, we apply a downsampling operation to $X^{\prime }$ to obtain Y. Subsequently, the downsampled feature passes through a depth-wise convolution, which produces the query (Q), key (K), and value (V) representations defined as follows:

(6) $$Y=AvgPool(X^{\prime })$$

(7) Q=DWConv(Y),K=DWConv(Y),V=DWConv(Y)

Subsequently, we compute the similarity between the queries and keys to obtain the channel attention matrix Attn. A normalization operation is then applied to Attn, and the result is used for weighting:

(8) $$\begin{array}{c} Attn=\frac{QK^{T}}{\sqrt{V}}\\ {Attn}_{s}=Softmax\left(Attn\right)\\ {Attn}_{out}=V{Attn}_{s} \end{array}$$

Finally, the channel attention output is combined with the spatially enhanced feature to obtain the final output feature:

(9) $$Y^{\prime }={X^{\prime }Attn}_{out}$$

### 3.3. Cross-Stage Partial Shifted Pinwheel Mixed Convolution

**Figure 4:**
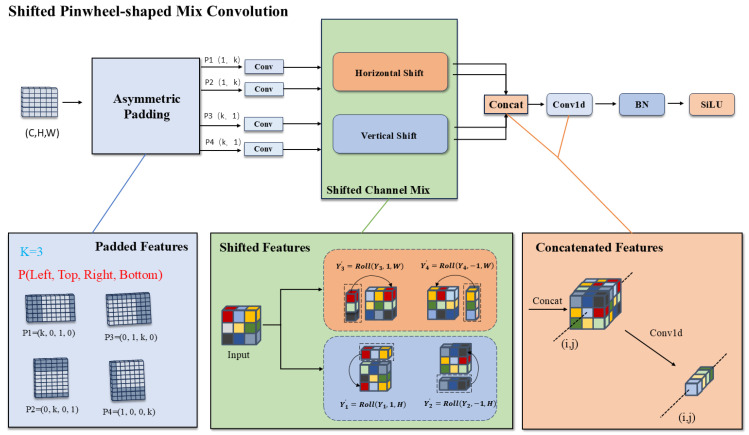
Architecture of the SPMConv module. P (Left, Right, Top, Bottom) denotes asymmetric padding to the left, right, top, and bottom, respectively. Horizontal Shift and Vertical Shift denote the horizontal and vertical channel shifts within the Shifted Channel Mix module.

**Figure 5:**
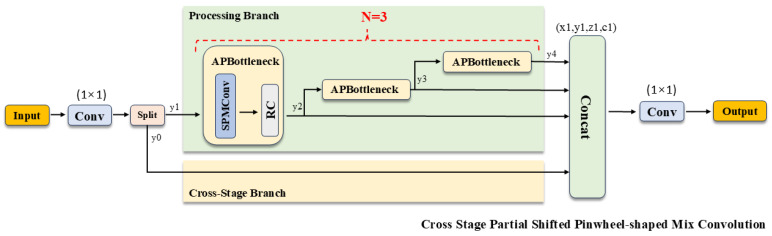
Architecture of the CSP-SPMConv module.

**Figure 6:**
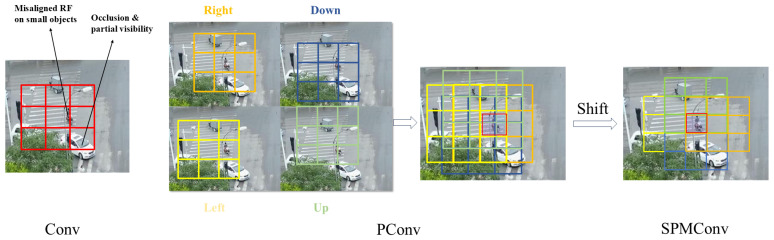
The visualization diagram of the spatial misalignment in aerial images processed by SPMConv.

Drawing inspiration from the morphological similarities between infrared small targets and distant aerial objects, we introduce the Shifted Pinwheel-shaped Mix Convolution (SPMConv) and its advanced variant, CSP-SPMConv. As illustrated in Figure 4 and Figure 5, the core design leverages asymmetric padding to capture orientation-specific features, effectively matching the blob-like distribution of small targets. To mitigate the spatial misalignment inherent in aerial imagery, we introduce a ‘Shift’ mechanism (Shift Channel Mix) to enhance the network’s capability for dynamically adjusting the receptive field center. As illustrated in Figure 6, targets under standard convolution often reside on the periphery of the sampling grid. Since edge pixels typically carry lower weights than central pixels during convolution, this results in attenuated feature extraction for small objects, causing them to be overwhelmed by background noise.While PConv utilizes asymmetric padding to extend the receptive field in various directions, it fails to fundamentally resolve the issue of spatial feature divergence. In contrast, SPMConv leverages the shift mechanism to rectify this scattering issue. It empowers the network to dynamically recalibrate the receptive field center with zero parameter overhead, thereby effectively alleviating spatial misalignment. Specifically, the Shift Channel Mix cyclically shifts feature maps to capture contextual information from the four cardinal directions. It then geometrically realigns the spatial neighbors (top, bottom, left, right) of the central pixel to the same coordinate (i,j) along the channel dimension, enabling the subsequent 1×1 convolution to efficiently aggregate the divergent local spatial context. Crucially, we embed this mechanism within a Cross-Stage Partial (CSP) architecture [32]. This design choice is pivotal: it not only optimizes gradient flow during backpropagation but also aggregates low-level spatial details with high-level semantics, achieving a superior balance between detection accuracy and computational efficiency. The specific implementation formulas are provided as follows:

SPMConv serves as the core processing unit for CSP-SPMConv. Initially, we apply asymmetric padding to the input tensor $X\in R^{c_{0}\times h_{0}\times w_{0}}$, where $c_{0},\;h_{0},\;\mathrm{and}\;w_{0}$ denote the channels, height, and width of *X*, respectively. To enhance stability, the convolutional operation subsequently utilizes Batch Normalization (BN) and the SiLU activation function.

(10) $$\begin{array}{c} Y_{1}=SiLU\left(BN\left(P_{\left(k,0,1,0\right)}\left(X\right)⊗W_{cw}^{1\times k}\right)\right),\\ Y_{2}=SiLU\left(BN\left(P_{\left(0,k,0,1\right)}\left(X\right)⊗W_{cw}^{1\times k}\right)\right),\\ Y_{3}=SiLU\left(BN\left(P_{\left(0,1,k,0\right)}\left(X\right)⊗W_{ch}^{k\times 1}\right)\right),\\ Y_{4}=SiLU\left(BN\left(P_{\left(1,0,0,k\right)}\left(X\right)⊗W_{ch}^{k\times 1}\right)\right) \end{array}$$

Here, $P_{\left(k,0,1,0\right)}$ denotes the padding parameter, where the subscript (k,0,1,0) corresponds to the padding values for (left, top, right, bottom), respectively. In the figure, *k* is set to 3. The symbol ⊗ represents the convolution operator. $W_{cw}^{1\times k}$ denotes the 1×k kernel used for horizontal convolution, and $W_{ch}^{k\times 1}$ denotes the k×1 kernel used for vertical convolution.

Subsequently, we apply the Shift Channel Mix operation to the features resulting from the parallel convolutions. Specifically, $Y_{1}$ and $Y_{2}$ are shifted downwards and upwards by 1 pixel along the height (H) dimension, respectively. Concurrently, $Y_{3}$ and $Y_{4}$ are shifted right and left by 1 pixel along the width (W) dimension, respectively.

(11) $$\begin{array}{c} Y_{1}^{\prime }=Roll\left(Y_{1},1,H\right),Y_{2}^{\prime }=Roll\left(Y_{2},-1,H\right),\\ Y_{3}^{\prime }=Roll\left(Y_{3},1,W\right),Y_{4}^{\prime }=Roll\left(Y_{4},-1,W\right) \end{array}$$

Then, the shifted features are concatenated and fused using a 2×2 convolution kernel.

(12) $$Y_{cat}=Cat\left(Y_{1}^{\prime },Y_{2}^{\prime },Y_{3}^{\prime },Y_{4}^{\prime }\right)$$

(13) $$f_{SPC}\left(X\right)=SiLU\left(BN\left(Y_{cat}⊗W_{cat}^{k\times 1}\right)\right)$$

Finally, a residual connection is established between the output of the SPConv module and the input feature *X* to form the APBottleneck module shown in the structural diagram.

(14) $$f_{APBottleneck}X=X+fSPC\left(X\right)$$

Regarding the CSP architecture, the initial input feature $X_{i}\in R^{B\times C\times H\times W}$ is first fed into a 1×1 convolutional layer. This serves to adjust the channel dimensions, thereby facilitating the subsequent feature splitting. Here, $y_{0}$ and $y_{1}$ represent the split features, and $W^{1\times 1}$ denotes the 1×1 convolution kernel.

(15) $$y_{0},y_{1}=\mathrm{Split}\left(\mathrm{SiLU}\left(\mathrm{BN}(X_{i}⊗W^{1\times 1})\right)\right)$$

Subsequently, we feed the separated features $y_{0}$ and $y_{1}$ into two distinct branches for processing: the Processing Branch and the Cross-Stage Branch.This design facilitates the learning of higher-level semantic information while simultaneously reducing model complexity.The Processing Branch is composed of *N* APBottleneck modules, while the Cross-Stage Branch does not perform any processing.

(16) $$\begin{array}{c} y_{2}=f_{APBottleneck,1}\left(y_{1}\right),\\ \vdots \\ y_{n+1}=f_{APBottleneck,n}\left(y_{n}\right) \end{array}$$

Finally, feature aggregation and fusion are performed.

(17) $$y_{\mathrm{out}}=\mathrm{SiLU}\left(\mathrm{BN}\left(\mathrm{Cat}(y_{0},y_{1},\ldots ,y_{n},y_{n+1})\right)\right)$$

## 4. Experiments

### 4.1. Dataset

**VisDrone2019**: This dataset comprises 10,209 aerial images captured by UAVs under diverse weather conditions and from various viewing angles. It encompasses ten predefined categories: pedestrian, car, truck, bus, tricycle, bicycle, motorcycle, stroller, occluded person, and other. The dataset partitioning, detailing the training, validation, and test sets, is presented in Table 1.

**UAVVaste** [33]: This dataset is a low-altitude small-target dataset for garbage detection. It contains 772 UAV aerial images, with a total of 3718 annotation boxes, all labeled as one category: “rubbish”.

**UAVDT** [34]: This is a challenging aerial dataset characterized by complex scenarios, comprising 40,735 images with a primary focus on vehicle targets. In this study, we select it as a supplementary benchmark to validate the generalization capability of SCA-DEIM in traffic surveillance scenarios.

**Figure 7:**
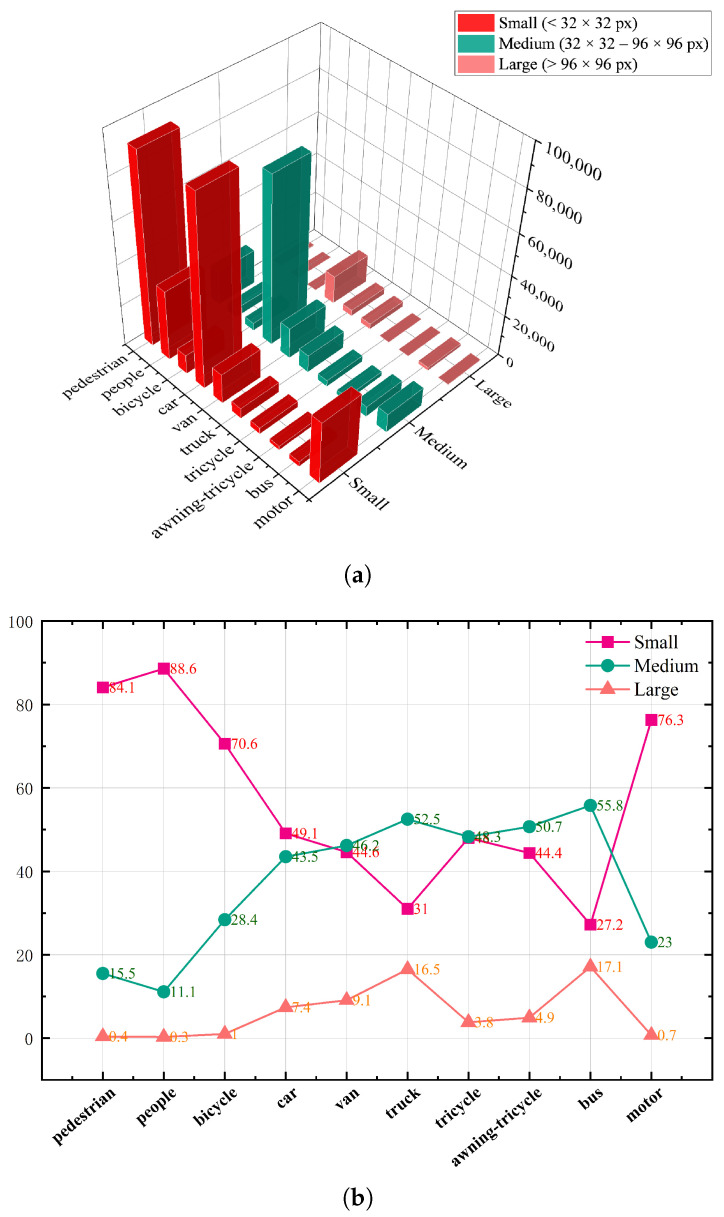
Distribution map of targets in various categories in the VisDrone2019 dataset. (**a**) The 3D bar chart of the size distribution of targets in each category in the VisDrone2019 dataset, where the X-axis represents category, Y-axis represents size, and Z-axis represents count. (**b**) Line graph of the percentage distribution of targets in each category by size in the VisDrone dataset.

To analyze the data distribution of VisDrone2019, we visualized the quantitative proportions of objects by class and by size. According to Figure 7, small objects constitute the majority of the dataset, accounting for 62.4% of the total instances. Among these, pedestrians (84.1%), people (88.6%), and motorcycles (76.3%) are predominantly small-sized objects, reflecting the characteristic of distant targets appearing small from a UAV perspective. Medium-sized objects account for 32.7% of the total, and are primarily concentrated in vehicle-related classes, such as cars (43.5%), vans (46.2%), trucks (52.5%), and buses (55.8%). Large objects are relatively scarce, comprising only 4.9% of the total instances. However, they constitute a higher proportion within the truck (16.5%) and bus (17.1%) categories.

This dataset is characterized by several challenging attributes: an extremely high proportion of small objects, complex background scenes, severe occlusions, and a high degree of overlap among different object classes. These attributes render the dataset particularly suitable for research focused on small object detection.

### 4.2. Evaluation Metric

To assess our approach’s performance in detection, a set of metrics for performance assessment was employed, including: Average Precision (AP), ${\mathrm{AP}}_{50}$, Average Precision for small, medium, and large objects (${\mathrm{AP}}_{s}$, ${\mathrm{AP}}_{m}$, ${\mathrm{AP}}_{l}$), Average Recall (AR), detection speed (FPS), as well as parameter count and computational complexity. These metrics are defined below.

The accuracy and completeness of the detection outcomes are quantified by Precision and Recall, respectively, which are formulated as:

(18) $$Precision=\frac{TP}{TP+FP},Recall=\frac{TP}{TP+FN}$$

Here, TP (True Positives) denotes the number of samples correctly predicted as positive, FP (False Positives) denotes the number of samples incorrectly predicted as positive (i.e., predicted as positive but actually negative), and FN (False Negatives) denotes the number of samples incorrectly predicted as negative (i.e., predicted as negative but actually positive). Mean Average Precision (mAP) represents the mean of AP scores computed over all object categories, serving as an indicator of a model’s overall detection capability.

(19) $$mAP=\frac{1}{N}\sum_{n=1}^{N}P\left(R_{n}\right)$$

Here, *N* is the number of categories and $AP_{n}$ is the Average Precision for the *n*-th class. ${\mathrm{AP}}_{50}$ denotes the AP when the Intersection over Union (IoU) threshold is set to 0.5. ${\mathrm{AP}}_{s}$, ${\mathrm{AP}}_{m}$, and ${\mathrm{AP}}_{l}$ represent the AP for small, medium, and large objects, respectively. Average Recall (AR) represents the average of recall values across different IoU thresholds, defined as:

(20) $$AR=\frac{1}{K}\sum_{k=1}^{K}{Recall}_{k}$$

where *K* is the number of different IoU thresholds selected for the calculation.

### 4.3. Experimental Setup

In this study, DEIM-D-Fine is employed as the baseline model. All experiments were conducted on an NVIDIA GeForce RTX 3080Ti (12 GB) GPU. Input images were resized to a uniform resolution of 640×640. The model was trained using the AdamW optimizer with a weight decay of $1\times 10^{-4}$ an initial learning rate of $3\times 10^{-4}$, and a batch size of 4. The entire training process spanned 180 epochs, as detailed in Table 2.

### 4.4. Comparison of Different Attentions in the Backbone Network Feature Extraction Stage

To evaluate the impact of different attention mechanisms on feature extraction, we conducted a comparative analysis of four modules: ECA, CBAM, SCSA, and our proposed ASCSA. These modules were integrated into all four stages (Stages 1–4) of the feature extraction backbone. Building upon this, we further compared the performance metrics of ASCSA when integrated into all backbone stages (Stages 1–4) versus its integration into only the high-level semantic feature extraction stages (Stages 3 and 4). The experimental results are presented in Table 3.

The experimental results indicate that the ECE attention mechanism [35] employed in the baseline outperforms the ECA and CBAM mechanisms for this specific task. This outcome is potentially because the ECA mechanism solely models channel attention without spatial modeling, whereas CBAM processes channel and spatial attention in series. This serial configuration may lead to information fragmentation and impede the effective extraction of fine-grained details, thereby resulting in suboptimal performance for small object detection. The results also show that ASCSA performs marginally better than SCSA. We attribute this to ASCSA’s ability, in this task, to maintain non-linear expressiveness while providing smoother gradient variations. This characteristic subsequently enhances its feature modeling capabilities, particularly for small objects and against complex backgrounds. A comparison of the two integration strategies revealed that while integrating ASCSA into all stages (Stages 1–4) yields a slight improvement in performance metrics over integrating it into only Stages 3 and 4, it nearly doubles the total parameter count. This implies a significant increase in both computational and storage overhead. We hypothesize that during the low-level semantic feature extraction stages (Stages 1 and 2), the primary objective is to maintain feature integrity and local stability. Overly sophisticated attention mechanisms, such as SCSA and ASCSA, might disrupt these fundamental patterns. Furthermore, the substantial increase in parameters leads to an imbalance between the acquired performance gains and the escalated computational complexity.

We conclude that applying ASCSA to all stages does not yield a desirable balance between accuracy improvement and computational overhead. Therefore, the module is integrated solely into Stages 3 and 4, which enhances performance without compromising efficiency, preserving the model’s lightweight characteristics.

To further validate the rationale behind our architectural design, we conducted comparative experiments on the activation functions within the ASCSA module. The goal was to verify that SiLU outperforms the Sigmoid function in facilitating spatial-channel synergy for small object detection. Specifically, we initialized the model with pre-trained weights and fine-tuned it for 50 epochs.

As presented in Table 4, replacing the standard Sigmoid (used in the original SCSA) with SiLU results in a significant boost in performance, particularly for small objects (${\mathrm{AP}}_{s}$). Unlike Sigmoid, which saturates at 1.0, the unbounded nature of SiLU allows the network to linearly amplify the response of weak UAV targets while preserving gradient flow in deep layers. This structural upgrade transforms the mechanism from passive masking to active feature recalibration.

### 4.5. Performance of Different Convolutional Modules in Feature Fusion

To further demonstrate the efficacy of the CSP-SPMConv module, we conducted comparative experiments evaluating the impact of different convolutional types and various asymmetric kernel sizes on overall performance, as shown in Table 5 and Table 6.

First, through comparative experiments using different convolutional variants, we can clearly observe the superiority of the CSP-SPMConv module. Specifically, the baseline model exhibits the lowest parameter count and computational complexity, yet it also delivers the poorest small-object detection performance (9.0%). When PConv is introduced, both the model parameters and computational cost increase, but this trade-off yields gains in AP and $AP_{s}$. In contrast, SPMConv equipped with the Shift Channel Mix achieves a far more notable improvement, with AP rising to 18.3% and $AP_{s}$ to 9.8%. This performance gain can be attributed to the mechanism’s enhanced ability to capture fine-grained semantics. When further integrated with the CSP architecture, the model reaches the optimal balance, attaining the highest $AP_{s}$ performance of 10.1% while simultaneously achieving a significant reduction in computational complexity, thanks to the CSP structure’s effectiveness in eliminating redundant computation.

Second, we investigated the impact of the asymmetric padding size on AP. As shown in Table 6, k=3 is identified as the optimal solution. When k<3, the receptive field may be insufficient to fully capture target information, leading to a decline in AP. Conversely, while an excessively high *k* expands the receptive field, it risks introducing background noise that distracts from the target, in addition to increasing parameters and computational complexity. Thus, a kernel size of 3 achieves the best balance between receptive field expansion and parameter efficiency.

### 4.6. Performance Comparison Experiment of Mainstream Methods

To demonstrate the effectiveness of SCA-DEIM, we selected several recent mainstream models for a comparative analysis. In the category of Real-time Object Detectors, we included the S and M versions of YOLOv11 [36], the S and M versions of YOLOv12 [37], and the S version of YOLOv13 [38]. For Object Detectors for UAV Imagery, the S and M versions of FBRT-YOLO were selected [39]. In the domain of End-to-end Object Detectors, our comparison included RTDETR-R18, the N and S versions of D-Fine, and the N and S versions of the baseline. Furthermore, to facilitate a comprehensive comparison across different model scales, we also trained an SCA-DEIM-S version.

Table 7 presents the comparative results between our method and current mainstream approaches on the VisDrone2019 dataset.

**Figure 8:**
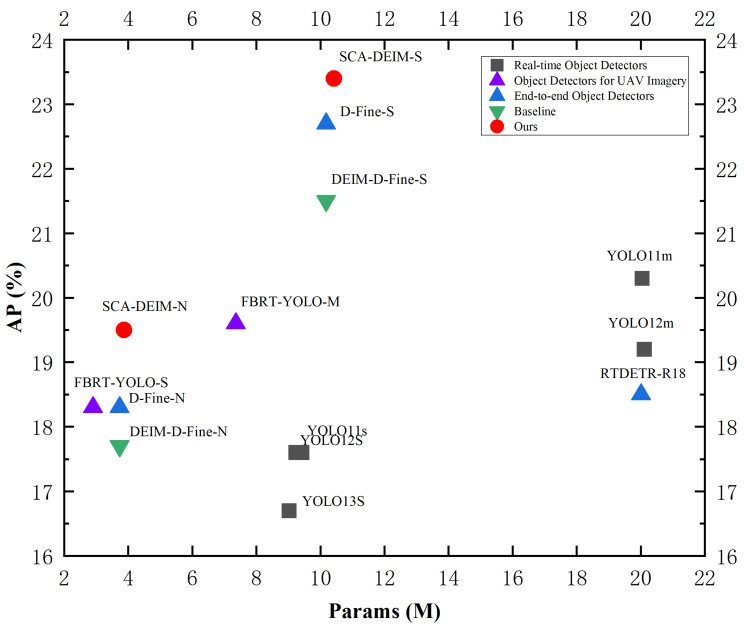
Performance scatter plot of different detection models on the VisDrone 2019 dataset.

To visually evaluate the efficiency of the proposed method, we compare the trade-off between parameter count (Params) and detection accuracy (AP) in Figure 8. As illustrated, the proposed SCA-DEIM series (marked in red) resides in the upper-left corner of the plot, indicating a superior balance between model complexity and performance. Compared to the Baseline and recent real-time detectors such as YOLOv11 and YOLOv12, SCA-DEIM achieves significantly higher AP while maintaining a compact model size, validating its effectiveness for resource-constrained UAV applications.

As shown in Table 7, YOLOv12-M exhibits rather outstanding performance metrics within the YOLO series. However, SCA-DEIM-N surpasses its performance. Specifically, compared to YOLOv12-M, although SCA-DEIM-N achieves only a 0.3% increase in average precision (AP), it obtains significant 1.9% and 1.0% boosts in small object precision (${\mathrm{AP}}_{s}$) and large object precision (${\mathrm{AP}}_{l}$), respectively, while possessing only 20% of the parameters of YOLOv12-M. Compared to FBRT-YOLO-M, SCA-DEIM-S achieves increases of 3.8% in AP and 4.5% in ${\mathrm{AP}}_{s}$, with GFlops at only 42% of those of FBRT-YOLO-M. Compared to RT-DETR-R18, SCA-DEIM-S improves AP by 5.1% and AP50 by 5.3%, while remaining on par for ${\mathrm{AP}}_{s}$. Most notably, the parameter count of SCA-DEIM-S is only half that of RT-DETR-R18, and its computational complexity is also significantly lower. When compared against the D-Fine and DEIM-D-Fine series, SCA-DEIM demonstrates significant improvements in both AP and ${\mathrm{AP}}_{s}$.

Compared to the baseline, SCA-DEIM-N achieves increases of 1.8% in AP, 1.7% in ${\mathrm{AP}}_{50}$, 2.3% in ${\mathrm{AP}}_{s}$, and 2.0% in ${\mathrm{AP}}_{l}$. SCA-DEIM-S achieves increases of 1.9% in AP and 1.7% in ${\mathrm{AP}}_{s}$, respectively, and our method also reduces computational complexity by 0.6 GFlops compared to the baseline. The experimental results demonstrate that our method is more suitable for the task of small object detection and achieves an effective balance between accuracy and lightweight design. This further validates the effectiveness of our proposed modules in cross-scale feature modeling and information interaction, offering a novel approach to the design of lightweight object detection models.

We selected the UAVVaste and UAVDT datasets to further verify the robustness of our approach in different urban environments, as shown in Table 8 and Table 9. Experimental results indicate that SCA-DEIM-N achieves the highest AP and best small object detection ($AP_{s}$) in both sparse and dense scenarios. This confirms the superior generalization and robustness of our method, proving its adaptability to diverse detection tasks.

**Figure 9:**
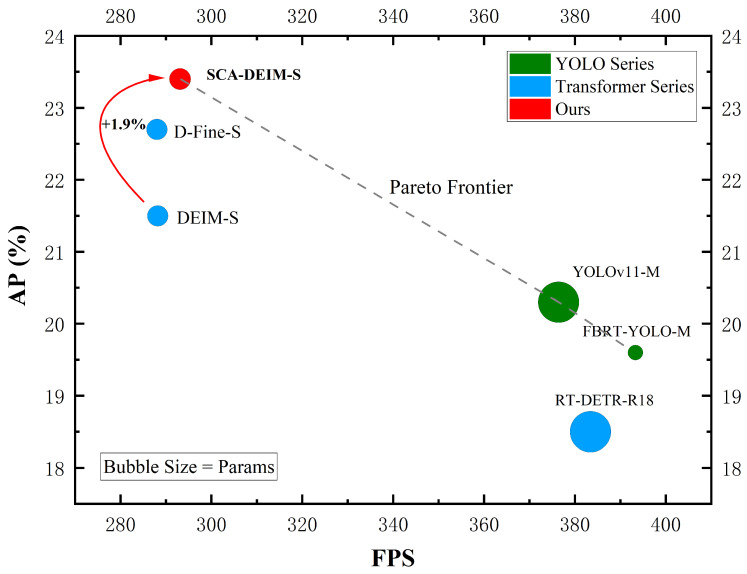
Speed–accuracy trade-off comparison on the VisDrone2019 dataset.

To evaluate the deployment feasibility of SCA-DEIM in high-performance aerial computing scenarios, we conducted a comprehensive inference speed analysis on an NVIDIA GeForce RTX 3080Ti GPU. All models were accelerated using TensorRT 10.11.0 with FP16 precision and a batch size of 1. Figure 9 illustrates the speed–accuracy trade-off, where the bubble size corresponds to the parameter count. As observed, the proposed SCA-DEIM-S (marked in red) establishes a new Pareto frontier within the high-accuracy regime. Specifically, compared to the baseline, our method achieves a substantial accuracy improvement of +1.9% AP while maintaining a rapid inference speed of approximately 295 FPS, effectively balancing computational cost and detection performance. While the YOLO series exhibits higher inference throughput, SCA-DEIM-S demonstrates superior capability in capturing small and occluded targets. This justifies a marginal compromise in speed for significant precision gains, which are paramount for safety-critical missions.

Complementing the server-side analysis, we further extended our benchmark to the NVIDIA Jetson Orin Nano (4 GB) to rigorously validate the practical deployment capabilities of SCA-DEIM in embedded UAV scenarios. Serving as a representative resource-constrained edge platform, this device imposes significantly stricter latency and memory constraints than server-based environments. To address these challenges, all models were exported to ONNX format and optimized via TensorRT 8.6.2 in FP16 mode. Inference performance was measured with a batch size of 1 to accurately simulate real-time processing conditions.

Table 10 details the inference efficiency on the NVIDIA Jetson Orin Nano. SCA-DEIM-N achieves a high throughput of 54.6 FPS with an ultra-low latency of 18.3 ms, satisfying the strict real-time requirement (<33 ms) for UAV navigation. Compared to YOLOv11-S, our model substantially reduces computational overhead, cutting parameters by 58.9% and GFLOPs by 66.8%, while maintaining comparable responsiveness. Furthermore, relative to the baseline, SCA-DEIM-N reduces computational complexity and improves inference speed despite a negligible increase in parameters, demonstrating a superior trade-off between efficiency and accuracy.

The above experimental results demonstrate that the proposed method is more suitable for small object detection tasks, achieving an effective balance among detection accuracy, lightweight design, and inference speed. Moreover, these results further validate the effectiveness of the proposed modules in cross-scale feature modeling and information interaction, offering a novel perspective for the design of UAV-based object detection models.

### 4.7. Ablation Experiment

**Figure 10:**
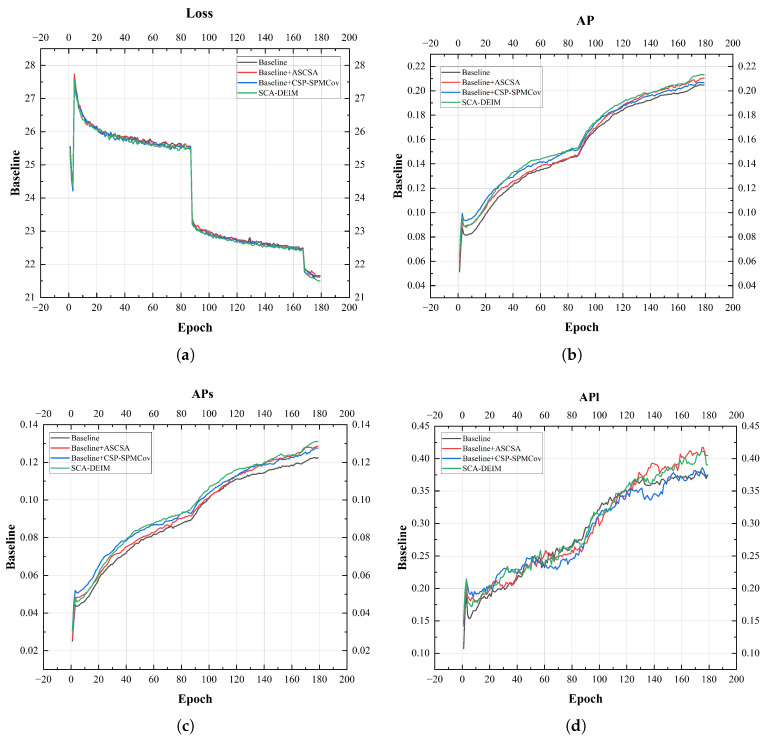
Performance metric curve of the model. (**a**) represents the Loss curve graph, (**b**) represents the AP curve graph, (**c**) represents the ${\mathrm{AP}}_{s}$ curve graph, (**d**) represents the ${\mathrm{AP}}_{l}$ curve graph.

To validate the effectiveness of ASCSA and CSP-SPMConv, a series of ablation experiments were carried out on the VisDrone dataset with four model configurations: A, B, C, and D. Here, A serves as the baseline; B represents the baseline augmented with ASCSA (Baseline + ASCSA); C represents the baseline augmented with CSP-SPMConv (Baseline + CSP-SPMConv); and D is the complete SCA-DEIM model. Table 11 presents the comparative results of this ablation study, and Figure 10 displays the corresponding training curves.

As depicted in Figure 10, the AP metrics experience a rapid increase by the 88th epoch, which marks the end of the second stage. This is precisely the intended outcome of enabling the Mosaic and Mixup techniques during this phase. Mosaic operates by randomly stitching four different training images into a new, composite image, applying random scaling, cropping, and arrangement during the process. Mixup linearly interpolates two images at a specific ratio, while simultaneously mixing their corresponding labels (i.e., bounding boxes) and class probabilities at the same ratio. The synergy of these two methods significantly enhances sample diversity, thereby improving the detection model’s generalization performance, convergence stability, and robustness.

Table 11 and Figure 10 illustrate that integrating the ASCSA module improves the detection performance, yielding increases of 1.1% in AP, 1.2% in ${\mathrm{AP}}_{s}$, and 1.2% in ${\mathrm{AP}}_{l}$ over the baseline. This indicates that ASCSA effectively enhances semantic feature extraction capabilities through its spatial-channel synergistic attention mechanism. Concurrently, the introduction of the CSP-SPMConv module increased AP by 0.3% and ${\mathrm{AP}}_{s}$ by 1.1%, validating the effectiveness of its multi-scale feature fusion and partial-feature-sharing design for fine-grained feature extraction. When both modules were integrated, all performance metrics reached their peak values. This strongly demonstrates that ASCSA and CSP-SPMConv have a synergistic relationship in semantic feature extraction and multi-scale feature fusion, collaboratively enhancing the model’s detection accuracy for both overall and small objects.

**Figure 11:**
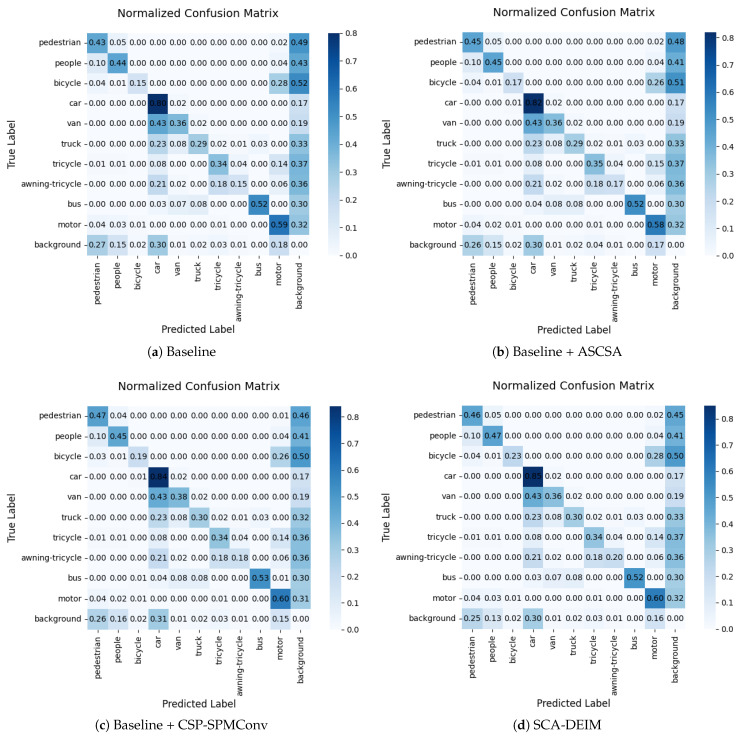
Comparison of confusion matrices between the baseline and our proposed method.

Figure 11 illustrates the normalized confusion matrices from the ablation study on the VisDrone2019 validation set. The diagonal elements represent the classification accuracy for each category, while the off-diagonal elements indicate the misclassification rates. It can be observed that with the individual introduction of the ASCSA and CSP-SPMConv modules, the values along the diagonal generally increase, whereas the off-diagonal values decrease. Notably, when these two modules function synergistically, the detection accuracy for targets such as “pedestrian”, “bicycle”, and “car” is significantly enhanced, effectively reducing their corresponding misclassification rates.

Furthermore, we observed that when trained for 180 epochs under the same strategy, the complete SCA-DEIM model achieved a lower training loss compared to the other configurations. This suggests that the fusion of the ASCSA and CSP-SPMConv modules not only enhances detection accuracy but also facilitates a more stable and efficient optimization process. The lower loss value further validates that the proposed modules can effectively guide the network toward faster convergence and superior feature learning capabilities.

## 5. Visualization

**Figure 12:**
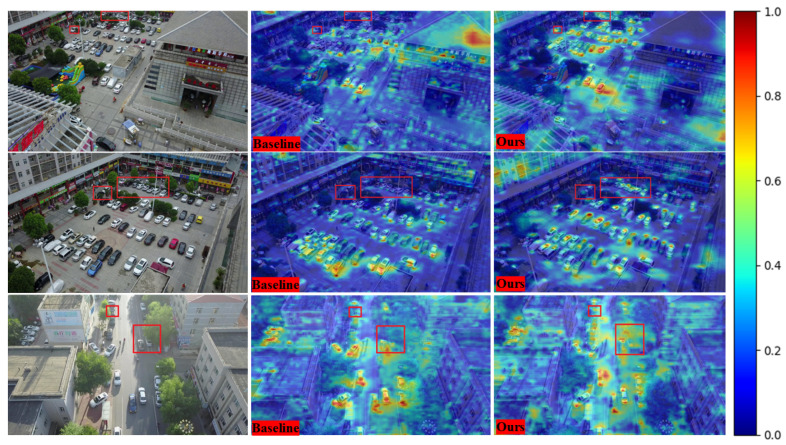
Heatmaps of SCA-DEIM and the baseline model on the VisDrone2019 dataset.

In Figure 12, we present heatmap visualizations of the model on the VisDrone dataset, with regions containing small and occluded objects specifically highlighted. Analyzing these three samples, we observe that our method (SCA-DEIM) outperforms the baseline model in localizing small and occluded targets. The heatmaps further indicate that the baseline exhibits weak localization for distant and occluded objects and fails to accurately capture small targets at close range.

In contrast, SCA-DEIM significantly enhances the heat activation values for these distant small and occluded targets, demonstrating stronger robustness. This indicates its superior capability to capture target features in these highly challenging scenarios. Furthermore, SCA-DEIM effectively improves the localization of small targets at close range, mitigating the missed detection (false negative) problem observed in the baseline model, particularly in high-density regions where targets overlap.

Under high-illumination conditions, the baseline model frequently exhibits over-suppression or omission of small targets, especially in high-light regions (e.g., reflective surfaces or overexposed areas). Conversely, SCA-DEIM significantly ameliorates these issues, successfully detecting small and occluded targets even in high-light environments. Notably, the model stably maintains high localization accuracy under complex illumination conditions, such as high contrast or overexposure.

**Figure 13:**
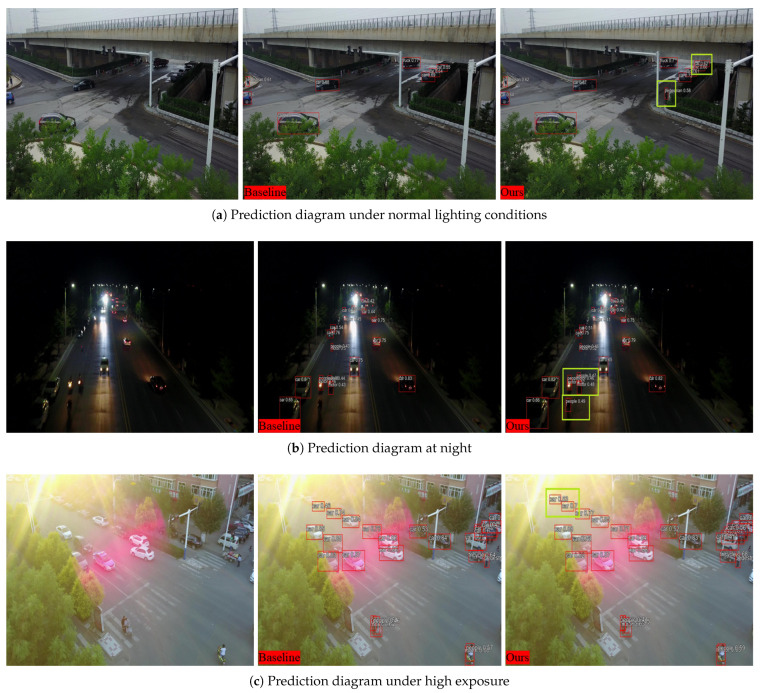
Prediction bounding box diagrams of SCA-DEIM and the baseline on VisDrone2019 under different lighting conditions.

To further evaluate the model’s performance under varying environmental conditions, this paper presents a qualitative comparison of prediction bounding boxes in daytime, nighttime, and high-light scenarios. These visualizations illustrate the detection results of both the baseline model and SCA-DEIM in these settings. The differing results are highlighted using green boxes, as depicted in Figure 13.

Figure 13a displays the prediction results in a daytime environment. The visualization reveals that the baseline model suffers from missed detections (false negatives), particularly for small objects and distant occluded targets. In contrast, SCA-DEIM generates an increased number of prediction boxes, successfully identifying the small and occluded objects that the baseline failed to detect.

Figure 13b illustrates the predictions in a nighttime environment. It is observable that the baseline model exhibits significant missed detections under low-light conditions, failing to correctly identify a portion of distant small and occluded targets. Conversely, the SCA-DEIM model demonstrates substantially enhanced detection capabilities in this setting. Not only does it produce a markedly higher number of prediction boxes with fewer missed detections, but it also achieves more accurate detection of distant small targets, such as vehicles and pedestrians. This indicates the model’s improved robustness in dark and shadowed environments.

Figure 13c presents the predictions in a high-light environment. It is evident that even under conditions of high exposure, SCA-DEIM continues to ameliorate the baseline model’s tendency to miss small and occluded targets.

**Figure 14:**
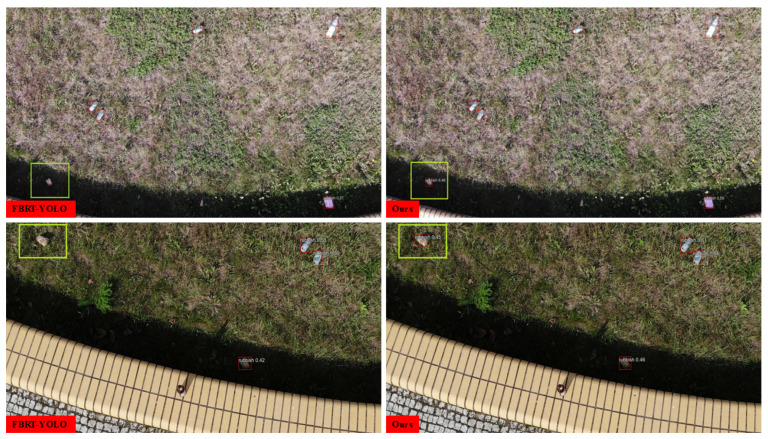
Prediction framework of SCA-DEIM and mainstream methods on UAVVaste.

Figure 14 presents a comparison of prediction result diagrams between SCA-DEIM and the mainstream method FBRT-YOLO on the UAVVaste dataset. We have highlighted the differences with green bounding boxes. As can be seen from the figure, SCA-DEIM can effectively capture small garbage targets and avoid missed detections. Additionally, we observe that the detection accuracy of other small targets in the figure is also improved to a certain extent compared with FBRT-YOLO. This indicates that in practical applications, SCA-DEIM achieves favorable performance in scenarios such as garbage inspection and urban environment monitoring.

**Figure 15:**
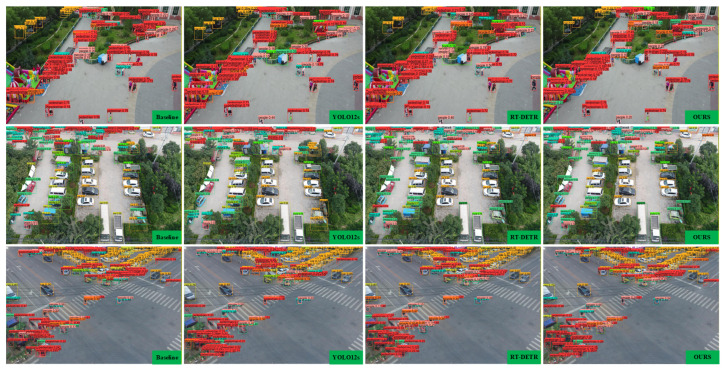
Qualitative comparison of detection results on the VisDrone2019.

To intuitively validate the superiority of SCA-DEIM, we present a qualitative comparison with YOLOv12-S and RT-DETR in Figure 15. The rows from top to bottom correspond to three distinct scenarios: dense crowds, parking lots, and intersections. In the dense crowd scenario (first row), YOLOv12-S and the Baseline suffer from severe missed detections regarding overlapping pedestrians. In contrast, SCA-DEIM successfully discriminates individuals within the crowd, demonstrating the effectiveness of the ASCSA module in enhancing fine-grained feature representation. In the parking lot and intersection scenarios (second and third rows), the comparative methods fail to detect distant vehicles and small objects at the image edges. However, SCA-DEIM accurately captures these targets. This robustness is attributed to the CSP-SPMConv module, which aligns receptive fields and enforces cross-channel interaction, thereby significantly reducing the miss rate for small and occluded objects compared to RT-DETR and YOLOv12-S.

## 6. Conclusions

This paper presents SCA-DEIM, a lightweight and real-time object detection framework tailored for Unmanned Aerial Vehicle (UAV) imagery. To address the critical challenges commonly encountered in aerial scenes—such as weak feature responses, spatial misalignment, and severe occlusions—we introduce two novel components into the DEIM architecture. First, the Adaptive Spatial and Channel Synergistic Attention (ASCSA) module is integrated into the backbone to enhance the capture of subtle features, thereby improving high-level semantic representation and spatial context modeling. Second, the Cross-Stage Partial Shifted Pinwheel Mixed Convolution (CSP-SPMConv) is designed to enable efficient multi-scale feature fusion, significantly boosting the perception of small and occluded objects while alleviating spatial misalignment.

Comprehensive experiments conducted on the VisDrone2019, UAVVaste, and UAVDT datasets demonstrate that SCA-DEIM achieves a superior balance between detection accuracy and model complexity. The results indicate that the proposed method not only delivers substantial improvements in small-object detection compared with the baseline but also maintains strong robustness under various illumination conditions, including low-light and high-exposure environments.

For future work, we plan to deploy SCA-DEIM on embedded UAV platforms to evaluate its real-time inference performance in practical flight missions. Additionally, we aim to explore advanced model compression techniques—such as network pruning and knowledge distillation—to further reduce computational overhead, making the method even more suitable for edge-computing scenarios with limited hardware resources.

## Figures and Tables

**Table 1:** VisDrone 2019 dataset.

Dataset	Quantity
Train	6471
Validation	548
Test	1610
Total	10,209

**Table 2:** Training Strategy on the Dataset.

Stage	Epoch	Training Strategy
1	1–4	Basic data augmentation (e.g., cropping, flipping) was applied, and a learning rate warmup strategy was enabled.
2	4–88	Mosaic and Mixup data augmentations were enabled.
3	88–168	A linear learning rate decay was adopted, and the intensity of data augmentation was progressively reduced.
4	169–180	Data augmentation was completely disabled.

**Table 3:** Performance of different attention mechanisms in feature extraction.

Model	Params	GFLOPs	AP (%)	AP_50_ (%)	AP*_s_* (%)	AP_*m*_ (%)	AP*_l_* (%)
Baseline	3.73 M	7.12 G	17.7	32.2	9.0	26.2	37.6
Baseline + ECA	3.60 M	7.23 G	16.9	31.4	8.3	26.1	37.2
Baseline + CBAM	3.88 M	10.43 G	17.1	31.7	8.1	25.4	36.0
Baseline + SCSA	7.28 M	20.83 G	18.2	33.0	9.5	26.8	38.1
Baseline + ASCSA (all)	7.31 M	20.91 G	18.8	34.1	10.2	27.2	40.2
Baseline + ASCSA (S3–S4)	3.70 M	7.06 G	18.6	33.6	10.0	26.4	38.8

**Table 4:** Comparison of different activation functions in the ASCSA module.

Activation	Mechanism	AP (%)	AP_s_ (%)
Sigmoid	Bounded Gating (0, 1)	14.0	5.8
ReLU	Hard Threshold	13.6	5.4
**SiLU (Ours)**	**Unbounded Recalibration**	**13.8**	**6.2**

**Table 5:** Performance of Different Convolutional Modules in Feature Fusion.

Model	Params	GFLOPs	AP (%)	AP_50_ (%)	AP_*s*_ (%)
Baseline	3.73 M	7.12 G	17.7	32.2	9.0
Baseline + PConv	5.80 M	10.78 G	17.9	31.4	9.3
Baseline + SPMConv	5.68 M	10.93 G	18.3	30.7	9.8
Baseline + CSP-SPMConv	5.71 M	10.34 G	18.0	31.8	10.1

**Table 6:** Comparison Experiments of Asymmetric Padding Convolutional Kernels K in SPMConv.

Kernels (*K*)	Params	GFLOPs	AP (%)
1	3.53 M	7.98	16.9
2	4.38 M	9.01	17.4
**3 (Ours)**	**5.41 M**	**10.34 G**	**18.0**
4	6.02 M	10.95	17.8

**Table 7:** Performance comparison of different detection models on the VisDrone 2019 dataset. The evaluation metrics include model parameters (Params), computational complexity (GFLOPs), and detection accuracy (AP, AP_50_, AP_*s*_, AP_*l*_). Our method and the baseline are evaluated across three different seeds, with the reported values being the mean, and the numbers in parentheses indicating the standard deviation (sd).

Model	Publication	Input Size	Params	GFLOPs	AP (%)	AP_50_ (%)	AP_*s*_ (%)	AP_*l*_ (%)
**Real-time Object Detectors**								
YOLOv11-S	arXiv 2024	(640, 640)	9.42 M	21.3 G	17.6	31.3	8.0	36.4
YOLOv11-M	arXiv 2024	(640, 640)	20.04 M	67.7 G	20.3	35.0	9.8	41.3
YOLOv12-S	arXiv 2025	(640, 640)	9.23 M	21.2 G	17.6	31.2	8.1	35.6
YOLOv12-M	arXiv 2025	(640, 640)	20.11 M	67.2 G	19.2	33.6	9.4	38.6
YOLOv13-S	arXiv 2025	(640, 640)	9.02 M	20.1 G	16.7	29.7	7.7	38.7
**Object Detectors for UAV Imagery**								
FBRT-YOLO-S	AAAI 2025	(640, 640)	2.90 M	22.9 G	18.3	32.3	8.5	42.5
FBRT-YOLO-M	AAAI 2025	(640, 640)	7.36 M	58.7 G	19.6	34.4	9.4	42.1
**End-to-end Object Detectors**								
RT-DETR-R18	CVPR 2024	(640, 640)	20.01 M	60.0 G	18.5	33.3	13.9	42.3
D-Fine-N	ICLR 2025	(640, 640)	3.73 M	7.12 G	18.3	33.4	9.3	44.2
D-Fine-S	ICLR 2025	(640, 640)	10.18 M	24.86 G	22.7	39.4	12.8	46.8
DEIM-D-Fine-N	CVPR 2025	(640, 640)	3.73 M	7.12 G	17.7 (±0.33)	32.2 (±0.38)	9.0 (±0.29)	37.6 (±0.39)
DEIM-D-Fine-S	CVPR 2025	(640, 640)	10.18 M	24.86 G	21.5 (±0.37)	38.4 (±0.28)	12.2 (±0.24)	40.5 (±0.51)
**Ours**								
**SCA-DEIM-N**	–	**(640, 640)**	**3.87 M**	**7.06 G**	**19.5 (±0.27)**	**33.9 (±0.35)**	**11.3 (±0.31)**	**39.6 (±0.44)**
**SCA-DEIM-S**	–	**(640, 640)**	**10.42 M**	**24.57 G**	**23.4 (±0.24)**	**38.6 (±0.32)**	**13.9 (±0.28)**	**41.3 (±0.48)**

“–” indicates that the value is not reported in the source.

**Table 8:** Performance comparison of different detection models on the UAVVaste dataset.

Model	AP (%)	AP_*s*_ (%)	AP_*m*_ (%)
YOLOv11-S	27.1	26.7	45.3
FBRT-YOLO-S	38.3	30.4	57.2
RT-DETR-R18	36.3	34.3	59.1
DEIM-D-Fine-N	38.5	35.1	56.3
**SCA-DEIM-N**	**39.5**	**36.3**	**58.6**

**Table 9:** Performance comparison of different detection models on the UAVDT dataset.

Model	AP (%)	AP_50_ (%)	AP_*s*_ (%)
FBRT-YOLO-S	16.4	29.1	7.9
DEIM-D-Fine-N	15.5	27.8	7.1
**SCA-DEIM-N**	**17.2**	**30.3**	**8.3**

**Table 10:** Inference speed and latency comparison on NVIDIA Jetson Orin Nano (4 GB) with TensorRT (FP16). Lower latency indicates better real-time responsiveness.

Model	Params (M)	GFlops	FPS	Latency (ms)
YOLOv11-S	9.42	21.3	56.4	17.7
DEIM-D-Fine-N	3.73	7.12	53.5	18.7
**SCA-DEIM-N (Ours)**	**3.87**	**7.06**	**54.6**	**18.3**

**Table 11:** Ablation experiments on the VisDrone2019 dataset.

Model	Epoch	Params	GFLOPs	AP (%)	AP_*s*_ (%)	AP_*m*_ (%)	AP_*l*_ (%)
A	180	3.73 M	7.12 G	17.7	9.0	26.2	37.6
B	180	3.70 M	7.06 G	18.8	10.2	26.4	38.8
C	180	5.41 M	10.34 G	18.0	10.1	26.1	37.9
D	180	3.87 M	7.06 G	19.5	11.3	27.6	39.6

## Data Availability

The datasets utilized and analyzed in the present study are publicly accessible via the following repositories: VisDrone2019 (available at https://github.com/VisDrone/VisDrone-Dataset (accessed on 1 October 2025)), UAVVaste (available at https://github.com/PUTvision/UAVVaste (accessed on 1 October 2025)), and UAVDT (available at https://sites.google.com/view/grli-uavdt (accessed on 9 December 2025)).
